# Evaluation of Children Caries Risk Factors: A Narrative Review of Nutritional Aspects, Oral Hygiene Habits, and Bacterial Alterations

**DOI:** 10.3390/children9020262

**Published:** 2022-02-15

**Authors:** Andrea Butera, Carolina Maiorani, Annalaura Morandini, Manuela Simonini, Stefania Morittu, Julia Trombini, Andrea Scribante

**Affiliations:** 1 Unit of Dental Hygiene, Section of Dentistry, Department of Clinical, Surgical, Diagnostic and Pediatric Sciences, University of Pavia, 27100 Pavia, Italy; 2 “Mamme & Igieniste”, 24125 Bergamo, Italy; dr.annalauramorandini@gmail.com (A.M.); manuelasimonini@libero.it (M.S.); morittustefania3@gmail.com (S.M.); juliatrombini@hotmail.it (J.T.); 3 Unit of Orthodontics and Pediatric Dentistry, Section of Dentistry, Department of Clinical, Surgical, Diagnostic and Pediatric Sciences, University of Pavia, 27100 Pavia, Italy; andrea.scribante@unipv.it

**Keywords:** caries risk assessment, nutrition, oral health, dental caries, dental prevention, dentistry

## Abstract

Dental caries is one of the most common diseases—both in adults and children—that occurs due to the demineralization of enamel and dentine by the organic acids formed from bacteria present in dental plaques through anaerobic metabolism of dietary sugars. The aim of this article is to provide a guideline to assess the risk of caries by looking for the main factors involved. Literature research was performed for studies that analyzed the factors most involved in the development of child caries, such as poor oral hygiene, bad eating habits (or food disorders), and an alteration of the oral bacterial flora—with an increase of *Streptococci* spp., *Lactobacilli* spp., *Candida albicans*, *Cryptococcus neoformans*, and *Candida sake*. It is therefore essential to assess the risk of caries in children, based on the assessment of risk factors, in order to be able to establish preventive and/or therapeutic approaches that will reduce or stop the development of dental caries. The use of fluoride products, products made from casein phosphopeptide-amorphous calcium phosphate, substituted zinc biomimetic hydroxyapatite products, or products containing self-assembling oligopeptide SAP-P_11_-4 are useful. In terms of the clinical approach, a communicative approach should be added to learn about the eating habits and the oral hygiene habits of the child and parents; in addition, the use of a simple method to frame the factors involved, and subsequently establish the risk of carious lesions in the child, allows the reduction of the DMFT (Decayed Missing Filled Teeth) or ICDAS (International Caries Detection and Assessment System) index on large scales.

## 1. Introduction

Dental caries is one of the most common chronic conditions in childhood. About 50% of preschoolers in several countries have at least one carious lesion, and this has a negative impact on the quality of life for both the child and the family. Caries is regarded as a public health problem, the etiology of which is reflected in eating habits, the type of dental brush, and socioeconomic indicators [1,2]. As far as the socio-economic situation is concerned, there is evidence that children from economically vulnerable or low-educated families have a higher prevalence rate of dental caries. This condition may influence parents’ or guardians’ perception of children’s oral health, having less knowledge of factors associated with caries and the need for dental care, as well as less access to health services [3,4].

In more economically disadvantaged countries, there is, in fact, a greater incidence of caries in children. The economic crisis and the lack of public health programmes affect the availability of both food and oral hygiene resources. High values of DMFT (Decayed Missing Filled Teeth) in countries such as Israel, Syria, Perù, and Serbia are linked to the lack of free distribution of fluoride toothpastes and toothbrushes in the poorest communities, and, in Syria, there is the economic crisis that has caused an increase in sugar intake and an increase in the barriers that families face for the availability of fresh food and oral hygiene products; studies carried out in Perù also highlight poverty, sugar intake, and a low literacy rate, which all encourage the increase of ECC in children [5,6,7,8].

Although the key factors causing carious lesions in adults and children are similar, there are unique risk factors regarding the latter, probably because the oral microbial flora and host defense mechanisms are still in development. In addition, the surfaces of freshly eroded teeth may have hypoplastic defects that facilitate the accumulation of bacterial biofilms and an increased risk of caries [9]. 

A review of the literature conducted in Canada has highlighted the major risk factors involved: enamel hypoplasia, eating habits, difficulty brushing, caregiver influence, low income, and low level of education. In addition, it would appear that those who do not perform a first examination of the child within 24 months have a higher risk of developing carious lesions, since it would appear to be higher (38%) in the male sex [10,11]. 

The aim, therefore, was to evaluate the main risk factors of caries in children in order to reduce the incidence through managing the oral microbiota, reducing the ingestion of acidic foods and sugars, and motivating oral hygiene at home—to reduce the presence of *Streptococci* spp. and *Lactobacilli* spp. In fact, nutrition is implicated in the risk of carious lesions and the erosion of the enamel; the latter disease is on the rise and is caused by food or acidic substances, without bacterial involvement. Incorrect habits, such as eating disorders, together with the acidic pH of some foods, weaken the enamel and consequently make the tooth structure more fragile. In addition, diet components can contribute to the development of enamel defects, such as hypoplasia and fluorosis [12,13].

### Caries Assessment

From the analysis of the literature, it is known that carious lesions occur due to the demineralization of enamel and dentine by the organic acids formed from bacteria present in dental plaques through the anaerobic metabolism of dietary sugars [14]. 

One of the main barriers available to the body is undoubtedly saturated saliva, which contains calcium and phosphate at pH 7 and promotes the process of remineralization, or deposits minerals in the porous areas where the process of demineralization of enamel or dentine began. If the pH of the oral cavity remains high enough for sufficient time, this process may occur; however, if the acidity persists, the demineralization process progresses, thereby increasing the porosity of the enamel until the formation of a carious lesion [15,16]. 

This development requires sugar and bacteria, but it is certainly influenced by tooth susceptibility, bacterial profile, saliva quantity and quality, and the time when fermentable food carbohydrates are available for bacterial fermentation [15]. In light of this evidence, and of the multiple factors involved, it is important to be able to keep the oral cavity well cleaned, even in a newborn, with the help of wet lap pads with a physiological solution after every sucking, avoiding spoiled habits such as baby bottles with milk and/or juices or any sweetened substance before going to sleep, thus preventing a serious form of caries, ECC (baby bottle caries) [17]. Then, at the appearance of the first dental elements, you can introduce the use of the toothbrush with an ergonomic handle and a non-slip grip, and a round and small head with extra-soft bristles. It is important that the child becomes familiar with the toothbrush. In addition, it is good practice to use a fluorinated toothpaste or toothpaste that contains remineralizing principles, with parent supervision to guide the child in brushing for at least 2 min, 2 times a day; this check is useful at least up to 7–8 years. From 3 years onwards, a fully deciduous dentition should be used for a fluorinated toothpaste with 1000 ppm of fluoride, or always a toothpaste with remineralizing principles, to increase the appearance of the first permanent elements—up to 1450 ppm of fluoride [18,19,20]. As for the intake of fluoride, which is contained in the toothpaste, the American Dental Association recommends the use of a “smear”-sized portion of toothpaste for up to 3 years of age to avoid the risk of fluorosis (problem related to possible ingestion of toothpaste by the child), from which you can then use a pea-sized portion of toothpaste [21]. 

## 2. Materials and Methods

As caries is the most common non-communicable disease in the world, based on the evidence gathered, it is necessary to implement oral health education programmes in schools (both for children and parents), programmes aimed at the most vulnerable groups, and programmes for the training of health professionals; it is also important to pay attention to the consumption of sugar, a problem related to other systemic diseases such as diabetes, cardiovascular diseases, cancer, and obesity, and thereby seeking to promote oral and nutritional hygiene education programs in schools. Furthermore, having highlighted the relationship between socioeconomic conditions and caries, it is appropriate to integrate policy programmes that promote affordable access to care essentials for dental needs and affordable fluoride toothpastes [22]. 

The International Caries Classification and Management System (ICCMS^TM^) has provided clear guidance for an appropriate management plan, which can be customized to be preventive and can be adapted to caries risk through clinical examination, risk assessment, and personalised assistance planning. The first step is the assessment of patient risk factors related to caries, namely head and neck radiation, dry mouth, inadequate oral hygiene practices, insufficient exposure to topical fluoride, high frequency of intake of sugary foods and drinks, symptom-driven dental care, socioeconomic status, and mother’s caries experience. Subsequently, the intra-oral risks related to caries, such as hyposalivation, PUFA factors, the experience of caries, the presence of plaque, the presence of devices or restorations that favor the accumulation of plaque, and exposed radicular surfaces, are assessed; the evaluation for the presence of caries can then be carried out visually and can possibly be associated with an x-ray examination, using the ICDAS ranking system (International Caries Detection and Assessment System) [23] and assessing whether these lesions are active or inactive. The ICDAS system uses several codes: the first for healthy teeth (code 0) and the next two for caries limited to enamel, white stain / brown (codes 1 and 2). The following two categories (code 3 and 4) are considered to be caries that extend to exposed dentine-free enamel. The remaining two categories (codes 5 and 6) are considered as caries with exposed dentin. The third step involves the analysis of these first collected data to provide information on the probability of new lesions and for indications of the activity of the lesions highlighted in order to develop plans for the treatment and management of the caries [24], as shown in Table 1.

Numerous tools have been developed for caries risk assessment, such as Cariogram, CAMBRA, ADA, and AAPD. The first uses an algorithm to calculate the percentage of risk combined with a clinical opinion; the second for the calculation of risk uses the instructions and the “caries balance”, evaluating the clinical observations, preventive factors, biological and environmental risk factors, and the opinion of the health care professional; the third uses modules that contain clinical observations, preventive factors, and risk factors, as well as the last-mentioned method.

### 2.1. Focused Question

In order to provide a new guideline for health professionals for the assessment of caries risk, research was carried out on the main risk factors involved.

### 2.2. Elegibility Criteria 

First, we analyzed the studies published in English in accordance with the following inclusion criteria:

Type of studies. Case-control, cross-sectional, cohort, longitudinal studies, and clinical trials.

Type of participants. Participant with caries and ECC.

Type of interventions. Case-control, cross-sectional, cohort studies, and clinical trials that have evaluated the possible etiological factors involved in the development of caries.

Outcome type. Each variable included in the studies was taken into account. We included studies that have assessed risk factors for children’s caries (primary outcome: dietary factors, oral hygiene, and microflora) and possible preventive factors (secondary outcome).

We included in the second phase only those studies that met all the inclusion criteria, that is to say, the analysis of the selected studies according to the exclusion criteria: (I) studies where the authors had not reported at least one of the parameters chosen as outcomes; (II) studies performed on participants with concomitant systemic pathologies/treatments that could have affected outcomes; and (III) studies that have not analyzed the possible risk factors of children’s caries. 

### 2.3. Search Strategy 

The review is based on the research of studies in reference to the PICO model (Population: caries risk in pediatric patients; Intervention: review of nutritional risk factors, oral hygiene, and change in microbiological flora; Comparison: with all the studies in the literature that compared various risks in addition to those examined; Outcome: a proactive approach allows us to improve the quality of life of young patients, both from a nutritional and oral hygiene point of view, thereby reducing the caries risk factors and DMFT values. A correct intake of fluoride and substitutes, such as biomimetic hydroxyapatite, remains the chief preventive approach thus far.), which were identified through bibliographic research in electronic databases, and by examining the bibliography of articles, on Pubmed (MEDLINE) and Google Scholar. Initially, all the study abstracts were taken into consideration, which evaluated the possible risk factors involved in the development of children’s caries.

### 2.4. Research

We performed the search using the following keywords: “children caries”, “early childhood caries”, “children caries” AND “risk factors”, “early childhood caries” AND “risk factors”, “dental caries” AND “primary dentition” AND “risk factors”. There is no time limit on the date of publication of the study.

### 2.5. Screening and Selection of Articles

**Figure 1:**
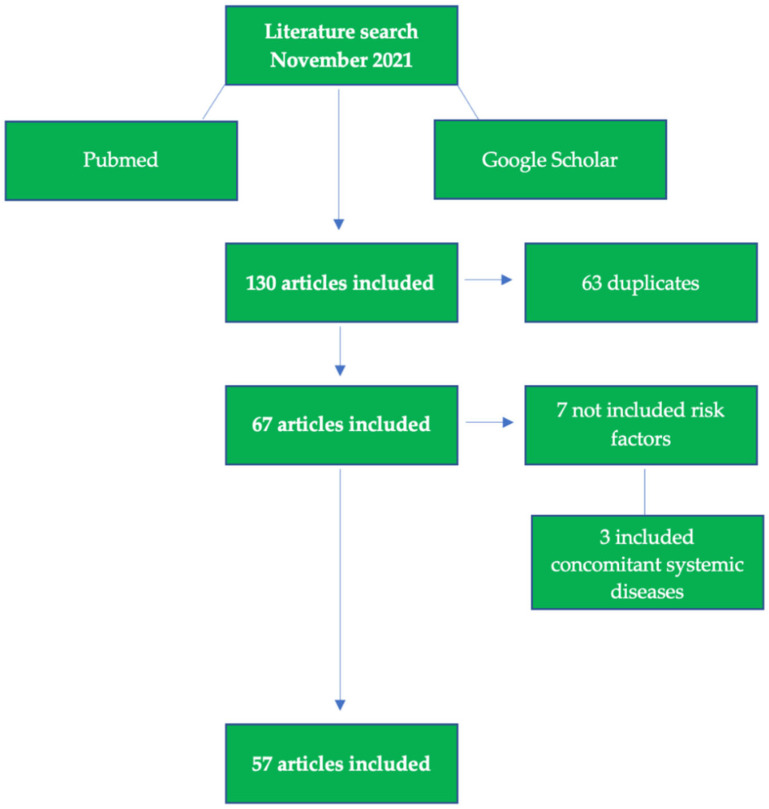
Flow chart of included studies.

The search produced 130 titles matching the search keywords and the information related to the inclusion criteria. The following flowchart shows the selection criteria used to select the final 57 articles that were used for the review analysis, shown in Figure 1.

## 3. Results 

The analysis of the studies included in the review (Table S1) revealed the main risk factors involved in carious lesions in children. 

The objective of this article was to provide guidance to dental professionals, based on the analysis of the risk factors related to the development of caries in children, such as eating habits, oral hygiene practices, and bacterial flora (Table 2). To this list the socioeconomic conditions and the level of education must surely be added, as well as the social policies—as is evident in the poorest countries. These indications, shown in Table 3, developed on the basis of the knowledge and indications already provided by ICCMS^TM^, are used to easily frame the risk of caries in children; from the collected data it is then possible to implement management and treatment plans. 

## 4. Discussion

On the basis of these considerations and a preliminary review of the literature, a table has been created of the main risk factors involved in the development of carious lesions in children and the associated caries risk in such a way as to facilitate healthcare professionals into the framework of all patients, while also encouraging a multidisciplinary approach with other health professionals, such as the pediatrician and the nutritionist, following the assessment of the child. 

The health professionals—namely dentists and dental hygienists—to whom the following table has been submitted (Table 3) have evaluated the simple and linear procedure, promptly managing to draw attention to all the risk factors, both local and systemic, on the basis of literature research for DMFT and caries activity, enamel defects and dental erosion, frequency of sugars and carbohydrates intake, saliva quantity and quality, remineralization (fluoride, substituted fluoride ad biomimetic hydroxyapatite and calcium phosphate, eating disorders, oral hygiene, socioeconomic status, and oral family health or other conditions), erosion by gastroesophageal reflux, and eating disorders in children, which are not frequent. In fact, among the 130 studies analyzed, some factors have been highlighted as strongly related to the development of caries in children, such as those related to diet, oral hygiene, changes in microbial flora (primary outcome of the review), breastfeeding, hypolasia, and socioeconomic factors. 

Among the risk factors involved in the development of carious lesions in children, poor oral hygiene, diet, and quality of oral bacterial flora have been recognized. 

Oral hygiene and its good practices should begin even before the eruption of the first element with the help of lap pads soaked in physiological solution, and then there should be the switch to the use of a toothbrush and fluorinated toothpastes with the help of parents. In this sense, ethnicity, social status, and degree of family education affect the development of injury to the hard tissue of the tooth. In fact, the prevalence of carious lesions is lower in children who brush their teeth more than once a day and especially those who brush before going to sleep with the help of parents. Children of school age do not have a regular brushing schedule in neither quantity nor quality [83]. Oral hygiene in children must be integrated from the first months of life in order to avoid plaque accumulations that can promote the onset of carious lesions over time. For this reason, it is essential to deal with a trusted dental team and pediatrician who can make the right indications based on the age of the child to maintain proper oral hygiene at home. Schools should also play a proactive role in the education of oral hygiene, seeking to provide programmes aimed at the age of the children and, therefore, relying on the proper health figures for reference.

As for the diet, it is also important for maintaining oral health and it is advisable to start to have good habits and good nutrition from when the child is a newborn. Milk is a complete food that is useful for the achievement and maintenance of oral health, given that it is a source of calcium, which is the basic constituent of the inorganic material of bones and the hard tissues of teeth; breast milk is the first food capable of supporting all nutritional needs, except for iron and vitamin C, until about the fourth month of life [84]. Subsequently, during growth it is preferable to eat foods of every type, preferring consistent foods that require good mastication to stimulate the salivary flow. It is desirable that the frequency of intake of fermentable carbohydrates should be reduced and limited to main meals, after which correct oral hygiene is possible [85]. Taking this type of food during the course of the day causes the repeated lowering of the pH, which promotes the demineralization of the hard tissues of the tooth, as well as the use of a sweetened pacifier and the non-nutritional use of a bottle containing sugary beverages. In this case, it is preferrable to use snacks based on fruit and vegetables, milk or cheese. Alternatively, low cariogenic sweeteners, which cannot be fermented, are sugar-alcohols (xylitol, sorbitol, and mannitol), synthetic sweeteners (aspartame, saccharin, cyclamates, and acesulfame-k), natural sweeteners (miracolin, taumatin, monellin, phyllodulcin, stevioside, and glycirryzin), hypo-acidogenic sugars, and hydrogenated derivatives of carbohydrates [86].

Another aspect that affects the development and predisposition of dental caries in children is the composition of oral bacterial flora, such as *Streptococcus mutans* and *Steptococcus sobrinus*, as well as *Lactobacilli* spp. (the latter exploits glucose-derived sucrose to destroy tooth tissue), appear to have an association with the development of enamel lesions as they metabolize sugar acids to produce acids, thereby contributing to the demineralization of the structure of dental enamel. The source for the presence of *Streptococcus mutans* already in newborns seems to be the transmission of the latter from the mother through saliva. In addition to these, other bacteria appear play a role in the beginning and progression of the lesion, such as species of *Actinomyces* and *Bifidobacterium* [87,88]. In addition, a study published in 2020 found other micro-organisms implicated in the progression of carious lesions: *Candida albicans* would appear abundant at the level of advanced lesions, along with *Streptococcus mutans* and *Scardovia wiggsiae*, while *Cryptococcus neoformans* and *Candida sake* seem to prevail in primitive lesions [89].

On the basis of these considerations, it is easy to see how important the multi-disciplinary approach between dental hygienists and dentists, pediatricians, and nutritionists is in ensuring an overall state of health of the child, orally and systemically. 

The importance of diet in children is clear given the repercussions it can have on oral health. It is advisable to give nutritional advice to better understand the eating habits of the whole family, with an on the sugar intake and the number of daily intakes of any food that contains simple carbohydrates. It is essential that there is an adequate introduction of energy and nutrients, such as vitamins, minerals, and calcium, which allow for the growth and formation of the organism; it is known, in fact, that there is a relationship between the consumption of fermentable sugars and the development of carious lesions. The danger from the frequency of consumption of these should be emphasized along with the fact that products such as fruit juices or sugary drinks favors childhood obesity. In Italy, according to data reported by the Istituto Superiore di Sanità (ISS), 20.4% of children are overweight and 9.4% suffer from obesity (including severely obese children, which represents 2.4%).

Having clarified which risk factors are mainly involved in the development of caries in children, Table S1 reports on the main problems to focus on to carry out a simple and possible analysis of the risk profile for caries in children. From this same table, and from the analysis of the literature, the relationship between the development of carious lesions and the feeding of the child is now clear. Once the risk profile has been analysed, it is then possible to implement targeted management and treatment plans for the child.

### Possible Prevention Approches

To date, the local administration of fluoride seems to be one of the most effective treatments in the primary prevention of carious lesions, as reported by the Guidelines of the Ministry of Health: *“The post-eruptive preventive effect of fluoride, obtained through the topical route of administration, is considered more effective than the pre-eruptive effect obtained through the systemic route of administration”; “The fluorinated toothpaste, therefore, represents a means of administration of primary importance in the prevention of caries”*. It is necessary, however, to pay attention to the composition of the toothpaste. In fact, not everyone releases enough local fluoride for the prevention of caries. It is necessary to distinguish free ionic fluoride, profluoride compounds, and fluoride compounds; the first type has the ability to interact with tooth enamel and perform anti-carious activity, the second type precipitates during brushing and releases ionic fluoride that can be effective in the prevention of carious lesions over time, and the third type has no efficacy in terms of prevention. Several studies have drawn attention to the effectiveness of fluorinated toothpastes and it has emerged that the use of fluorinated toothpaste, especially toothpaste containing at least 1500 ppm of fluoride, leads to a significant reduction in the development of carious lesions [90,91].

There are also other formulations available for the professional topical administration of fluoride, such as gels or varnishes, which are equally effective. Gels (with about 12,300 ppm of fluoride) are usually applied with the help of a mask, which is placed for about 4 min, while the paints (22,600 ppm), which are easier to apply and eliminate the risk of ingestion of fluoride by the child, are applied and brushed on the teeth clean and dry [92].

Other remineralizing systems used for primary prevention are those based on calcium phosphate, such as casein phosphopeptide-amorphous calcium phosphate, calcium amone phosphate, and bioactive glass containing calcium and sodium phosphosilicate [93]; studies have shown that the use of CPP-ACP (casein phosphopeptide-amorphous calcium phosphate) reduces the roughness of the enamel, which favors bacterial adhesion, thereby restoring and gradually repairing the central areas of the enamel [94].

Biomimetic mineralization from P_11_-4 is also known, in combination with the application of fluoride, as a non-invasive treatment for initial carious lesions thar is capable of regenerating enamel tissue and preventing the progression of lesions. This self-acting oligopeptide assembler is able to form a biological matrix, causing the regrowth of the crystals of dental minerals. This activity is demonstrated by studies in the literature, which show that primary carious lesions treated with P_11_-4 are significantly reduced [95,96,97].

Last but not least, hydroxyapatite products that are biomimetic zinc-substituted can be used for the remineralization of the enamel surface, such as toothpastes or mousse that is able to interact with biological tissues. The effectiveness has been demonstrated after one month of treatment with toothpaste, thanks to the deposition of calcium, phosphorus, and silicon ions, thereby forming a real coating layer on the surface of the enamel [98]; the mousse, instead, contains a high percentage of microrepair (30%) and is effective if applied daily for 10 days a month for about 10 min—already from the second cycle of application [99]. The use of these products in primary prevention would seem valid in the reduction of the risk for development of carious lesions in children, having antibacterial properties against *Steptococcus mutans* and being effective in the prevention of biofilm, based on the biomimetic coating they create on the surface of the enamel—even in children [100]. Remineralizing systems, if there is a low risk, and substances based on biomimetic zinc-substituted hydroxyapatite can be used to take proactive action to avoid the incidence of caries and, above all, an overdose of fluorinated substances, which in the long term can induce irreversible lesions. 

Table 4 summarizes the remineralizing products and their use for the risk management of caries in children. Finally, to date, non-pharmacological alternatives are available for those children who need chlorhexidine to counter possible gum inflammations, given by the accumulation of bacterial biofilms, such as probiotics—which are equally effective [101]. However, it may be used at 0.05% with the additional fluoride-based composition in the form of mouthwash for an extended period (not more than one month of treatment). 

## 5. Conclusions

Caries is a degenerative disease that affects the hard tissues of the tooth and is caused by the acidic action of bacteria that are normally present within the oral cavity. These bacteria proliferate in excess and take advantage of the presence of sugars introduced from the children’s diet. As can be seen from this report, it is a multifactorial etiology pathology, mainly linked to poor oral hygiene, bad eating habits, and an alteration of oral bacterial flora. There are, in fact, foods considered strongly cariogenic, such as sugars, sugary drinks, industrial snacks, caramel, candies, sweets, chocolate, industrial fruit juices, breakfast cereals, tomato preserves, sauces or balsamic vinegar, cured meats, canned bread, and many other foods that apparently do not contain sugar, but, in reality, have significant quantities. On the basis of the dietary habits of children and parents, caries risk should be established by taking into account all the relevant factors in order to provide prevention and/or maintenance protocols that are aimed at restoring the optimal conditions in the oral cavity by means of close professional hygiene sessions and remineralizing the areas of greatest risk by using products with fluoride or containing other remineralizing agents, such as biomimetic zinc-substituted hydroxyapatite.

## Figures and Tables

**Table 1:** Managing a patient’s risk factors (from ICCMS^TM^).

Low Risk	Medium Risk	High Risk
Tooth brushing 2/day with a fluoride toothpaste (≥1000 ppm), following the dental team instructions	Tooth brushing 2/day with a fluoride toothpaste (≥1450 ppm), following the dental team instructions	Tooth brushing 2/day with a fluoride toothpaste (≥1450 ppm), following the dental team instructions
	General Behaviour Modification in Oral Health	General Behaviour Modification in Oral Health
	Prescribed Fluoride mouth rinse	Prescribed Fluoride mouth rinse
Motivational engagement (discuss with patients how to improve oral health behaviours—including amount of sugar), maintain dental visits at risk-based intervals		
	Sealants Fluoride varnish 2 times/year Fluoride gel or solution Recalls up to every 3 months: professional cleaning and topical fluoride- application on active lesions	Sealants Fluoride varnish 2 times/year Fluoride gel or solution Recalls up to every 3 months: professional cleaning and topical fluoride- application on active lesions
	Motivational interviewing One-to-one dietary intake interventions Altering medication-induced hyposalivation Reducing the use of recreational drugs	Motivational interviewing One-to-one dietary intake interventions Altering medication-induced hyposalivation Reducing the use of recreational drugs
		Increase fluoride varnish to 4 times/year
		Topical fluoride application, counseling: reduce sugar amount and frequency

**Table 2:** Main risk factors involved in children’s caries.

Risk Factors Associated with Caries		
Dietary factors	High sugar consumptions (snacks, soft drinks, sugary bed-time drinks)	[25,26,27,28,29,30,31,32,33,34,35,36,37,38,39,40,41,42,43,44,45]
Oral hygiene	Frequency of toothbrushing, plaque accumulation	[36,37,38,40,41,44,45,46,47,48,49,50,51,52,53,54,55,56,57,58]
Presence of *Streptococcus mutans*		[29,59,60,61,62,63,64,65,66]
Breastfeeding	Up to 6 months	[40,50,55,67,68,69,70,71]
Hypoplasia		[38,72,73,74,75,76,77]
Socio-economic factors	Educational parental level, rural or urban domicile	[28,37,38,39,41,57,58,63,78,79,80,81,82]

**Table 3:** Determination of the caries risk.

Factors	Low Risk	Medium Risk	High Risk
DMFT and Caries activity	/	Within 24 months	Within 12 months
Enamel defects and dental erosion	/	On a few elements	Spread in both arcades
Frequency of sugars and carbohydrates intake	During the main meals	During main meals and in the morning and/or afternoon break	During main meals, in the morning and/or afternoon break and at night
Saliva quality and quantity	Abundant flow, high buffering capacity, low acidity	Medium flow, medium buffer capacity, medium acidity and medium bacterial charge	Low flow, low buffer capacity, high acidity and high bacterial load
Remineralization (fluoride, substituted fluoride as biomimetic hydroxyapatite and calcium phosphate)	Daily	Occasional	Absent
Eating disorders	/	/	Anorexia, bulimia, binge eating, gastroesophageal reflux
Oral hygiene (Silness & Loe plate index evaluation)	No bacterial stratification	Plaque along the gingival margin, free but biofilm interdental spaces visible to the naked eye	Plaque along the gingival margin and in the interdental spaces
Socioeconomic status and oral family health	High/no caries	Medium/low caries rate	Low/high caries rate
Special conditions	/	/	Asthmatic patients undergoing radiotherapy, with systemic pathologies, with orthodontic devices

**Table 4:** Indications for primary prevention in accordance with caries risk.

	Low Risk	Medium Risk	High Risk
**Primary prevention**	Oral hygiene instructions: 2 min of brushing, preferably after each main meal	Oral hygiene instructions: 2 min of brushing, preferably after each main meal	Oral hygiene instructions: 2 min of brushing, preferably after each main meal
	Collection of information on the eating habits and oral health of children and families	Collection of information on the eating habits and oral health of children and families	Collection of information on the eating habits and oral health of children and families
	**Professional oral hygiene every 6 months**	**Professional oral hygiene every 4 months**	**Professional oral hygiene every 3 months**
	Professional use of remineralizing agents: fluoride-based, casein phosphopeptide-amorphous calcium phosphate or zinc-substituted hydroxyapatite gel or mousse, self-assembling oligopeptide SAP-P_11_-4	Professional use of remineralizing agents: fluoride-based, casein phosphopeptide-amorphous calcium phosphate or zinc-substituted hydroxyapatite gel or mousse, self-assembling oligopeptide SAP-P_11_-4	Professional use of remineralizing agents: fluoride-based, casein phosphopeptide-amorphous calcium phosphate or zinc-substituted hydroxyapatite gel or mousse, self-assembling oligopeptide SAP-P_11_-4
	Home use of remineralizing agents, as toothpastes containing fluoride or zinc-substituted hydroxyapatite	Home use of remineralizing agents, as toothpastes containing fluoride or zinc-substituted hydroxyapatite and mousse with zinc-substituted hydroxyapatite once a day for 10 days, for about 10 min	Home use of remineralizing agents, as toothpastes containing fluoride or zinc-substituted hydroxyapatite and mousse with zinc-substituted hydroxyapatite once a day for 10 days, for about 10 min

## Data Availability

The data presented in this study are available on request from the corresponding author.

## Supplementary Materials

The following supporting information can be downloaded at: https://www.mdpi.com/article/10.3390/children9020262/s1, Table S1: Studies included.
