# Supplementary material for: Evaluation of Children Caries Risk Factors: A Narrative Review of Nutritional Aspects, Oral Hygiene Habits, and Bacterial Alterations
Source: Children (Basel). 2022 Feb 15;9(2):262. doi: 10.3390/children9020262 (PMC8870668; doi:10.3390/children9020262)
Supplement: Supplementary file 1 [file children-09-00262-s001.zip › children-1580871-supplementary.pdf]

Table S1: Studies included.

Article	Year	Problem	Intervention/Control	Outcome
**Is there a relationship between Birthweight and subsequent growth on the development of Dental Caries at 5 years of age? A cohort study**	2010	Examine the associations between childhood growth and the presence of dental caries at age 5.	There were enrolled 14 541 pregnancies, and a 10% sample of these were dentally examined and measured at 61 months of age. Birthweight was obtained from medical records, and birth length and birthweight were assessed by trained ALSPAC measurers. A number of social and lifestyle factors were treated as potential confounding factors. .	Of 985, children, 242 (24.6%) had caries at 61 months of age. After adjustment, increased weight at birth was associated with a small increased risk of caries at 61 months (OR: 1.08 (95% CI: 1.03, 1.13) per 100 g increase, P = 0.002). A similar association was noted with respect to increased length at birth. Current weight and height did not appear to be associated with caries risk. Children who had caries at 61 months had slower increases in weight and height between birth and 61 months than those without decay at 61 months.
**Randomized Clinical Trial of the Effect of Prental Fluoride Supplements in Preventing Dental Caries**	1997	Identifying the early determinants of risk for dental caries would be useful in its primary prevention.	1,400 women in the first trimester of pregnancy residing in communities served by fluoride-deficient drinking water were randomly assigned to one of two groups. During the last 6 months of pregnancy the treatment group received 1 mg fluoride daily in the form of a tablet and the control group received a placebo. Subjects were encouraged to use postnatal dietary fluoride supplements.	The study had sufficient power to detect an absolute risk reduction of 5.1% while only a 1.5% reduction was observed.
**Factors Associated with Early Childhood Caries Incidence among African-American Children in Alabama**	2015	To assess the relationships between different behavioral factors and Early Childhood Caries (ECC) in African-American pre-school children.	Data from an ongoing longitudinal study in Alabama was used and analyzed. The original "parent" study was designed to investigate the relationships between the acquisition of cariogenic bacteria associated with the eruption of first primary and permanent molars in high-risk African-American children and the pathogenesis of dental caries. In the first cohort (not the focus of	For those living in a non-fluoridated community, more frequent consumption of sweetened food, less frequent consumption of 100% juice, less frequent toothbrushing, and reporting a previous visit to a dentist were significantly associated with increased ECC incidence.

			this study), 91 children aged approximately five years at baseline were recruited, while in the second cohort (the focus of this study), 97 children aged 3–22 months were recruited over an 18-month period beginning in July, 2008, which was one year after the recruitment of children in the first cohort.	
**Dental caries patterns and oral health behaviors in Arizona infants and toddlers**	2001	Use tooth eruption sequence, and a tooth- and surface-specific caries analysis method to determine: (1) the temporal relationship between tooth eruption and caries onset; (2) the validity of pre-existing concepts of caries progression; and (3) the relationship of certain putative health behaviors with caries prevalence.	A total of 2428 Arizona children aged 6–36-months, who were recruited from WIC programs (a federal program for low-income children at nutritional risk), health fairs and private day care centers, received visual dental caries examinations. Additionally, an oral health behavior survey was administered to the parents of the 1529 children recruited from the WIC programs.	Dental caries was a significant health issue for these children under 3 years of age, and factors other than bottle feeding may play an important role in its etiology. Prevention of caries in children under age three will depend on a better understanding of the etiology as well as improved access to care
**Factors Associated with Caries Increment: A Longitudinal Study from 18 Months to 5 Years of Age**	2010	Identifying the early determinants of risk for dental caries would be useful in its primary prevention.	All children at the age of 18 months in one health care area were invited in the screening test for mutans streptococci (MS) and their caretakers interviewed for the study purposes. The 366 children who met the inclu- sion criteria were clinically examined after 42 months	At the age of 5 years 8 6 months, in 79 (21.6%) children the 42-month caries increment was 10. In the multivariate regression analysis, the strongest correlation was found with MS detected in the oral biofilm. Of the reported background factors the blue-collar occupation of the caretaker, child's sugar use, night feeding, use of other thirst quenchers than water and father's reported poor oral health were significantly correlated with the child's caries increment
**Dietary habits related to caries development and**	2015	describe the dietary habits of infants	Children with caries at 2 and 3 years of age and	Although a great variation in

**immigrant status in infants and toddlers living in Sweden**		and toddlers living% Sweden with special reference to caries prevalence at 2 and 3 years of age and to immigrant status	immigrant children had, when they were 1 year old, consumed caries-risk products and been given nocturnal meals and sweetened liquid in a feeding bottle more often than caries-free 2- and 3-year-olds and non-immigrant children	dietary habits was found in infants and toddlers, the use of sugar-containing products is widespread in Sweden even in early childhood
**The frequency of mother-infant salivary close contacts and maternal caries activity affect caries occurrence in 4-year-old children**	1991	Establish that maternal caries incidence and salivary contacts with a child are connected with caries infection and immunity	Dental caries was investigated in 248 4-year-old children in the Lohja District Health Centre, Finland in relation to the postpartum caries incidence rate in their mothers and the frequency of salivary close contacts between mothers and babies when the latter were 7 months old. Dental caries occurred in 32% of the children.	The results of the study support the theory that maternal caries incidence and salivary contacts with a child are connected with caries infection and immunity. Variations in bacterial challenge via such factors before and during the eruption of teeth may modify caries development in the primary dentition.
**Caries management by risk assessment: A review on current strategies for caries prevention and management**	2015	Focus on the repair of hard tooth tissues using noninvasive strategies	CAMBRA was developed to promote the clinical management philosophy in which the caries disease process is managed following the medical model. This involves an evaluation of the etiologic and protective factors and the establishment of the risk for future disease, followed by the development of a patient-centered evidence-based caries management plan	Many factors such as bacteria, carbohydrate diet, and host response, cause initiation of dental caries, and its progression. Assessment of the caries risk of the individual patient is a critical component in determining an appropriate management strategy.
**Ethnicity, Location, Age, and Fluoridation Factors in Baby Bottle Tooth Decay and Caries Prevalence of Head Start Children**	1992	Compare BBTD and caries prevalence among Head Start children who are members of four ethnic groups in five southwestern States	The sampling process was a stratified random site selection; it was used to obtain data on 1,230 children. This number consti- tuted 3 percent of the children enrolled in Head Start in Public Health Service	BBTD was prevalent in approximately 24 percent and 15 percent of the total sample, depending on the severity criterion used. Native American childrenhadasignificantlyhigher(P<0.05) prevalence than Hispanic, white, and black subjects. Rural children had significantly higher (P<0.05) preva-

			Region VI (Ar- kansas, Louisiana, New Mexico, Oklahoma, and Texas)where the study was conducted.	lence of BBTD than non rural children for all ethnic groups except whites.
**The caries-inhibiting effect of a fluoride drop program: a 3-year study on Chinese kindergarten children**	1998	Evaluate the caries-inhibiting effect of a fluoride drops program in two kindergartens in Chengdu, Sichuan Province, a fluoride-deficient area.	For 3 years, participating children ingested in school 0.25 mg fluoride daily, in the form of drops for 2 to 3-year-olds and 0.5 mg for children age 3 years and older. These systemic fluoride supplements were made available for over 180 days each year and they were distributed by teachers who maintained attendance records. Annual examinations were conducted for 176 2-year-olds who had used the supplements in the kindergartens and 148 control children. After 3 years, 128 test and 112 control children remained in the study.	The results demonstrated a marked reduction in dental caries in the control group: test children had 2.21 decayed, missing, or filled teeth (DMFT)-versus 4.32 in the control group, a reduction rate of 48.84%. The mean DMFT increments over the 3-year period were 1.76 and 3.92, respectively. Over a 1- to 3-year period of supplement use, the group provided with the fluoride drops had consistently lower caries prevalence than the control group. The differences were statistically significant.
**Global oral health inequalities: dental caries task group--research agenda**	2011	The IADR Global Oral Health Inequalities Task Group on Dental Caries has synthesized current evidence and opinion to identify a five-year implementation and research agenda which should lead to improvements in global oral health, with particular reference to the implementation of current best evidence as well as integrated action to reduce caries and health inequalities	Translate existing evidence into routine clinical and public health practice; to tailor interventions to a more equal and person-centered preventive focus; and to reduce any social gradient in health. The sequence of priorities is to start with clinical and public health implementation of existing research findings, and, in parallel, to seek new basic science insights in key areas.	

		between and within countries.		
**Caries prevalence and bottle-feeding practices in 2-year-old children with cleft lip, cleft palate, or both in Taiwan**	1999	The purposes of this study were to investigate the caries prevalence in cleft lip, cleft palate, or both in children under the age of 2 years and to evaluate parental attitudes toward bottle-feeding, dental care, and their relationship to baby bottle tooth decay (BBTD) in Taiwan	One hundred twenty-three 2-year-old children (68 boys and 55 girls) with cleft lip, cleft palate, or both were selected for this study. A questionnaire that asked questions about knowledge of oral health, knowledge and beliefs about BBTD, children's feeding habits, children's dental care, and parenting attitudes toward children with clefts was completed by the parents or caretakers. Children were divided into bottle-feeding and non-bottle-feeding groups according to the questionnaire responses of parents or caretakers. Each child was examined with a dental mirror and explorer under focused flashlight using defs index to determine the presence of BBTD.	Children with clefts who took a bottle to bed showed an increased risk of developing BBTD. The parents or caretakers of bottle-fed children also showed a lack of motivation to perform regular preventive dental home care for their children. This suggests that oral health promotion programs should begin in infancy for children with clefts and their parents.
**The Clinical, Environmental, and Behavioral Factors That Foster Early Childhood Caries: Evidence for Caries Risk Assessment**	2015	Review recent best available evidence behind the factors that influence caries risk assessment and the validity of strategies to assess the caries risk of young children.	Moderate to weak evidence supports the following recommendations: (1) Children should have a caries risk assessment done in their first year (or as soon as their first tooth erupts) as part of their overall health assessment, and this should be reassessed periodically over time. (2) Multiple clinical, environmental, and behavioral factors should be considered when assessing caries risk in young children, including factors associated with the primary caregiver. (3) The use of structured forms, although most may not yet be validated, may aid in systematic assessment of multiple caries risk factors and in objective record-keeping. (4) Children from low socioeconomic status groups should be considered at increased risk when developing community preventive programs.	
**Risk Factors Associated with Carious Lesions in Permanent First Molars in Children: A Seven-Year Retrospective Cohort Study**	2020	Evaluate the risk factors associated with the occurrence of caries in permanent teeth (PT) and in the permanent first molar (PFM) seven	Children born in 2005 who were enrolled in a Community Dental Program were included. A total of 278 children were enrolled. Evaluated risk factors were parental caries experience,	A cariogenic diet (sweets and soft drinks), toothbrushing frequency of <1 a day, a presence of df-t (decayed and filled temporary teeth) score of >0, low educational level of the mother, and existence of MIH were associated with high DMF-T or DMF-M values ($p < 0.05$). We can conclude that the intake

		years after their eruption.	educational level of the mother, routine medications, systemic diseases, dietary habits, toothbrushing frequency, existence of molar incisor hypomineralization (MIH) in the PT, and caries in the temporary teeth (TT).	of sweets and soft drinks, toothbrushing frequency, the presence of caries in TT, and MIH in PT were the best predictors of the occurrence of caries in PT and PFM.
**Relationships between DEF, demographic and behavioral variables among multiracial preschool children**	1989	Suggest that children's dental health is related to certain practices begun during the infant and preschool years.	Specific interventions (e.g., early weaning, parent-assisted toothcleaning, and diets high in milk and low in sugar) will decrease the incidence of caries in children.	
**Risk indicators of early childhood caries (ECC) in children with high treatment needs**	2017	Determine risk indicators and their correlation among children with high caries prevalence and high treatment needs, in order to facilitate the development of targeted prophylaxis programs that would reduce future occurrences of ECC or at least positively influence the outcome	For this purpose, between 2010 and 2014 the parents of these children (n=82) were interviewed in the University Children's Hospital in Basel (UKBB) prior to the children's treatment under general anesthesia. The standardized questionnaire included questions regarding the age of the child, the mother's country of origin, the oral hygiene, and the drinking habits of the child.	The analysis shows that the high mean dmft/ dmfs values (dmft: 9.49 and dmfs: 26.35) correlated significantly with the geographic origin of the mother ($p<0.05$), the beginning of tooth brushing ($p<0.05$), lack of supervised tooth brushing ($p<0.01$), and nighttime consumption of sugar-sweetened beverages ($p<0.05$). In contrast, the duration of breastfeeding and prolonged use of a baby bottle (about 2.5 years) did not have a clear impact on high caries prevalence.
**Dental caries and their association with socioeconomic characteristics, oral hygiene practices and eating habits among preschool children in Abu Dhabi, United Arab Emirates - the NOPLAS project**	2018	Assess dental caries and their associations with socioeconomic factors, oral hygiene practices and eating habits among Emirati and non-Emirati children in Abu Dhabi, United Arab Emirates (UAE).	The stratified sample comprised children aged 18 months to 4 years recruited from 7 nurseries. The World Health Organization (WHO) decayed, missing and filled teeth index (dmft) was used to analyze the dental status of the children. Parents completed a questionnaire regarding demographics, food consumption and oral habits. The study was	A total of 186 children with a mean age of 2.46 years, of which 46.2% were Emirati, participated. Overall, 41% of the children had dental caries. The mean dmft±SD was 1.70 ± 2.81 with a mean ± SD decayed component (dt) of 1.68 ± 2.80 and mean ± SD filled component (ft) of 0.02 ± 0.19. Emirati children showed higher mean dmft, Plaque Index and Significant Carries Index values than non-Emirati children ($P < 0.000$). Low maternal education, rural nursery location, infrequent tooth-brushing, frequent consumption of high-sugar food items and Emirati

			approved by the Research Ethics Committee at Zayed University, UAE (ZU15_029_F)	nationality were factors significantly associated with dental caries.
**Early childhood caries update: A review of causes, diagnoses, and treatments**	2013	Establish causes and treatments for early childhood caries		The relationship between breastfeeding and ECC is likely to be complex and confounded by many biological variables, such as mutans streptococci, enamel hypoplasia, intake of sugars, as well as social variables, such as parental education and socioeconomic status, which may affect oral health.
**Snacking habits and caries in young children**	2010	Investigate the association between snacking and caries in a population at high risk of dental caries.	American preschool children (n = 1,206) were recruited in the offices of paediatricians. Data on sociodemographic characteristics, oral hygiene, breast-feeding, use of bottle and snacking were collected by questionnaire. Plaque presence, the number of teeth and their caries status (deft) were scored.	Multivariate partial least squares (PLS) modelling revealed plaque presence, lowest income, descriptors for tooth exposure time (number of teeth and age) and cariogenic challenge (total intake of sugar-containing snacks and chips/crisps, and chips intake with a sugar-containing drink) to be associated with more caries. These differences were also found in univariate analyses; in addition, children who continued breast-feeding after falling asleep had significantly higher deft values than those who did not.
**A best practices approach to caries management**	2014	Landmark publications, original research, and systematic reviews are analyzed and reviewed to form the basis for this shift in patient care related to caries disease	Caries management by risk assessment represents best practices and is an evidence-based model that focuses on treating and preventing disease at the patient level rather than a surgical/restorative approach at the tooth level.	Caries management by risk assessment has emerged as the new paradigm in patient care and represents an evidence-based, best practices approach with the potential for significant advantages over traditional methods.
**Risk factors associated with deciduous tooth decay in Iraqi preschool children**	2014	Define risk factors associated with deciduous TD (DTD) in Iraqi preschool children.	From the 1st June to 31st December 2012, a case-control study was carried out on 684 children under the age of 6 years who attended Al-Aulwyiah pediatric teaching hospital in Baghdad. Clinical examination and World Health Organization caries diagnostic criteria	The mean DMFT score in the case group was 2.03 ± 1.39, of which decayed teeth formed 1.93. Males had a higher mean DMFT (2.10 ± 1.08) than females (1.96 ± 1.70) but with no statistically significant difference. The study revealed that residence, SES, parental education level and tooth brushing frequency were dependent risk factors significantly associated with DTD. However, gender, parental smoking and pattern of feeding during

			for decayed, missing and filled teeth (DMFT) were applied. Data including gender, residence, socio-economic status (SES), parental education level, parental smoking, tooth brushing frequency, type of feeding during infancy and the presence of any systemic disease in the child were sought	infancy were not significantly associated with DTD. Only four children with systemic disorders (1.2%), namely asthma and congenital heart diseases, were noticed to have DTD.
**Sociobehavioral factors associated with caries increment: a longitudinal study from 24 to 36 months old children in Thailand**	2014	Investigate sociobehavioral risk factors from the prenatal period until 36 months of age, and the caries increment from 24 to 36 months of the child in Thailand	The total sample size recruited was 783 infants. The sample size with dental caries data was 603 and 597, at 24 months and at 36 months, respectively. The sample size of having two assessment points with a dental examination (at 24 months and at 36 months) was 597.	Results indicate that the caries increment was 52.9%, meaning from 365 caries free children at 24 months 193 had developed dental caries at 36 months. The prevalence of dental caries was 34.2% at 24 months (n = 206) and 68.5% at 36 months of age (n = 409). In bivariate analysis, higher education of the mother, lower household income, bottle feeding of the infant, frequent sweet candy consumptions, and using rain or well water as drinking water were associated with dental caries increment, while in multivariate conditional logistic regression analysis lower household income, higher education of the mother, and using rain or well water as drinking water remained associated with dental caries increment.
**Apgar score and dental caries risk in the primary dentition of five year olds**	2010	Apgar score predicted dental caries in the primary dentition	A retrospective cohort study conducted in 2003 examined associations between conditions at birth and early life with dental caries experience at five years. Dental examination data for a random sample of five-year-old South Australian children were obtained from School Dental Service electronic records. A questionnaire mailed to the parents obtained information about neonatal status at delivery (five-minute Apgar score, birthweight, plurality, gestational age)	Of the 1398 sampled children with a completed questionnaire (response rate=64.6%), 1058 were singleton term deliveries among whom prevalence of dental caries was 40.1%. In weighted log-binomial regression analysis, children with an Apgar score of <=8 relative to a score of 9-10 had greater probability of dental caries in the primary dentition after adjusting for sociodemographic and behavioural covariates and water fluoridation concentration (adjusted PR=1.47, 95% CI=1.11, 1.95).

			and details about birth order, weaning, and behavioural, familial and sociodemographic characteristics.	
**Association of caries increment in preschool children with nutritional and preventive variables**	2015	Evaluate the influence of various risk factors on dental caries increment in deciduous teeth of preschool children over 2.5 years.	A longitudinal study was carried out in kindergartens in two German counties in Northern Hesse. At baseline examination in 2006-2007, the mean age of the children was 3.5 years. Caries experience was recorded according to WHO standards. Information about feeding practices during early childhood and preventive measures were collected by a structured questionnaire for each child.	Three hundred ninety-five (69.8%) of the 566 children showed no caries increment. A caries increase of 1 to 10 dmf-teeth was exhibited by 171 children (30.2%). The mean dmf-t increment amounted to 0.75. The bivariate analysis revealed that the consumption of sugary food and beverages had a negative impact on oral health. Early start of toothbrushing, use of fluoridated children's toothpaste, and frequent toothbrushing exerted a positive influence on dental health. Stepwise backward logistic regression analysis confirmed that a high social status has a significant positive impact on dental health ($p = 0.028$), whereas the consumption of sugary food and beverages was significantly associated with a higher dental caries increment ($p = 0.004$).
**Risk factors for severe early childhood caries in children younger than 4 years old in Beijing, China**	2008	Compare cariogenic factors and acidogenic ability of bacteria between severe early childhood caries (S-ECC) and caries-free children in Beijing, China.	The study consisted of 117 S-ECC children and 129 caries-free children < 4 years old. A questionnaire was designed to collect background information, feeding habits, and oral hygiene practices. Dental plaque samples were collected to test acidogenic ability of bacteria.	Compared with the mothers of caries-free children, those of S-ECC children had a lower education level and poorer knowledge of oral hygiene ($P < .05$). Night-feeding and eating sweets several times a day were significantly more common in S-ECC children than in caries-free children ($P < .001$). Forty-six S-ECC children but only 2 caries-free children received prechewed food ($P < .001$). The results of the Cariostat test showed that the majority of the caries-free children (81%) were at a low risk level, and most of the S-ECC children (78%) were at a high risk level ($P < .001$).
**Caries prediction model in pre-school children in Riyadh, Saudi Arabia**	1998	Evaluate the significance of variables such as oral hygiene, dietary habits, socio-economic status and medical history of a child in	A sample of 446 Saudi pre-school children, 199 males and 247 females, with a mean age of 4.13 years, were selected at random from clinics and schools. Selection was limited to subjects who	There was a highly significant difference between the two groups in: debris index ($P < 0.0001$), aged child started tooth brushing, ($P < 0.0001$), age breastfeeding was stopped ($P < 0.005$), nocturnal bottle feeding with milk formula ($P < 0.001$), use of sweetened milk ($P < 0.0001$), frequency of use of

		assessing the level of caries risk and to generate a caries prediction model for pre-school Saudi children.	either had no caries (dmft = 0) or who had high caries experience (dmft > 8). Each child was examined for caries experience and oral hygiene status. Their mothers were interviewed through a standardized questionnaire for information about oral hygiene habits of the children, diet history, childhood illness and socio-economic status.	soft drinks (P < 0.0005), frequency of consumption of sweets (P < 0.0001), and age at first dental visit (P < 0.0001). A caries prediction model developed through stepwise multivariate Logistic Regression (LR) analyses showed debris index, use of sweetened milk in bottle, frequency of consumption of soft drinks, frequency of intake of sweets and child's age at the first dental visit to be significant. Predictive probability of the model was 86.31% with a sensitivity of 90.1% and a specificity of 80.6%.
**Dental caries and related factors--a longitudinal study of Finnish immigrant children in the north of Sweden**	1990	Identify baseline variables that could predict caries	In this study the development of dental caries was observed in Finnish immigrant children between 5 and 8 years of age.	Dental health in this group of Finnish immigrant children was already very poor at the age of 5 and deteriorated further up to 8 years of age, when only 4% of the children were caries free. In the last three years, 48% had participated in an individualized prevention programme (average 0.6 hours). Using the parents' dental status as a predictor gave a higher percentage of correct classifications than the use of dmfs.
**Dental caries in pre-school children: associations with social class, toothbrushing habit and consumption of sugars and sugar-containing foods. Further analysis of data from the National Diet and Nutrition Survey of children aged 1.5-4.5 years**	1999	Examine the relative significance of dietary sugars, toothbrushing frequency and social class as predictors of caries experience (caries vs. no caries) among 1,450 British pre-school children who took part in the National Diet and Nutrition Survey.	This cross-sectional survey was based on a representative sample of children aged 1.5-4.5 years studied in 1992/3. Children were classified into four groups according to social class and toothbrushing habit. Diet/caries associations were examined for biscuits and cakes, sugar confectionery, chocolate confectionery and soft drinks, and the percentage of energy from non-milk extrinsic sugars, using data on amount and frequency of consumption from 4-day weighed dietary records.	The association of caries with sugar confectionery (both in amount and frequency) was only present among children whose teeth were brushed less than twice a day. Toothbrushing frequency appeared to have a stronger impact on caries prevention in non-manual compared with manual children. Household expenditure on confectionery was associated with caries only among children from the manual group.

**Prediction of dental caries development in 1-year-old children**	1995	Evaluate the predictive ability of variables studied in 1-year-old children that could be used to identify children at risk for early caries development.	Dietary habits, oral hygiene, fluoride exposure and occurrence of mutans streptococci were studied in 1-year-old children (n = 786) as well as the socio-economic and immigrant background of their parents.	In a multivariate logistic regression analysis, the variables significantly associated with caries at 3.5 years of age were immigrant background (p < 0.001), mother's education (p < 0.001), consumption of sugar-containing beverages (p < 0.001), mutans streptococci (p < 0.05) and candy (p < 0.05). The probability of caries development was 87% when all the variables associated with caries were present at 1 year of age. The relative risk (odds ratio) of those children to develop manifest caries at 3.5 years of age was estimated to be 32 times higher than in the children where corresponding risk factors were not present.
**Early childhood caries and risk factors in rural Puerto Rican children**	1998	Identify risk factors of early childhood caries	A sample of 167 Puerto Rican children whose ages ranged from six months to forty-seven months (mean = 23 months) were studied. Children were examined for Early Childhood Caries with light and mirror and a structured interview was administered to parents and caretakers to identify risk factors.	Results indicated that only 37.4 percent of the children were free of decalcification lesions or frank decay. While 53.9 percent of the children had lesions on maxillary incisors, 40.0 percent had molars affected by decalcification lesions and caries. Analysis of risk factors indicate that giving the baby a bottle when crying at night, number of adults and children in the family, use of fluoridated dentifrice, and age of the child were associated with the caries process in these children.
**Risk Factors of Early Childhood Caries Among Preschool Children in Eastern Saudi Arabia**	2021	determine the risk factors of early childhood caries (ECC) among preschool children from eastern Saudi Arabia	241 carers and their children from 10 daycares were recruited and asked to complete a questionnaire aimed at assessing their sociodemographic profile and oral health-related behaviors. This was followed by a dental examination of the children by two calibrated dentists	The mean dmft of the children was 4.39 (SD ± 4.25). According to logistic regression analysis, children who were bottle-fed between 7 and 12 months (Adjusted Odds Ratio (AOR): 0.110) or breastfed between 13 and 18 months (AOR: 0.028) were less likely to have ECC than those with a prolonged feeding duration (>18 months). Also, those with smoking carers (AOR: 0.176) were less likely to have ECC than those with nonsmoking carers. On the other hand, children who had working carers, mainly mothers, in the education sector (AOR: 11.105), were more likely to have ECC ($p < 0.05$). The risk factors associated with the presence of ECC among preschool children in eastern Saudi Arabia include the mother's occupation and the carer's

				smoking status, as well as feeding practices (particularly bottle feeding).
**Factors related to dental health in 12-year-old children: a cross-sectional study in pupils**	2005	identify factors related to the prevalence of caries in 12-year-old schoolchildren.	A cross-sectional study was carried out using a representative sample (n = 1217) of the population of 12-year-old schoolchildren in Galiza (northwest Spain). Independent variables were measured through a questionnaire, and dependent variables were determined through oral examination. Multiple and logistic regression were applied	The decayed, missing and filled permanent teeth/decayed, filled primary teeth (DMFT-dft) value in the sample was 1.83 (95% confidence interval [CI], 1.67-1.98), the DMFT value was 1.53 (95% CI, 1.37-1.67), and the prevalence of caries was 61% (95% CI, 57.7-64.5). The prevalence of caries was directly related to a low frequency of brushing, greater use of toothpaste, and a higher consumption of sweets
**Risk Factors associated with Early Childhood Caries**	2017	analyse factors associated with the susceptibility of early childhood caries (ECC), populations with a high risk of ECC were screened and guidance for ECC prevention was proposed.	A total of 392 children aged 24 to 71 months were selected for oral examination in Qingdao. Parents or guardians of the participants completed the questionnaires and decayed missing filled surface (dmfs) were recorded. Differences in caries condition and oral health behaviour in different families were compared. Risk factors related to ECC were screened. The subjects were finally grouped based on the obtained dmfs into three groups: caries-free, ECC and S-ECC (severe ECC)	There were significant differences among the caries-free, ECC and S-ECC groups in three parameters: eating too many sweets each day, brushing before and after sleeping, and whether parents helped to brush ($P < 0.01$). Combined factors such as the parents' level of education, oral health knowledge, attitudes, the family's annual income, the age of children when they start to brush and not brushing regularly were also significantly related to ECC ($P < 0.05$). No significant differences were observed among the three groups for these factors, including birth condition and nursing state, physical condition of the mother during pregnancy, feeding situation, if a pacifier was used during sleep, duration of brushing, frequency of mouth rinsing after meals each day and brushing with fluoride toothpaste ($P > 0.05$).
**Pattern and severity of early childhood caries in Southern Italy: a preschool-based cross-sectional study**	2014	Investigate prevalence and severity of early childhood caries (ECC) in a sample of children in Southern Italy and to identify factors that may be related to this condition.	The study was designed as a cross-sectional survey. The study population (children aged 36-71 months) attending thirteen kindergartens was randomly selected through a two-stage cluster sampling procedure. Parents/guardians of all	515 children participated in the study. 19% had experienced ECC, and 2.7% severe-ECC (S-ECC), with a mean dmft and dmfs scores of 0.51 and 0.99, respectively. Mean dmft was 2.68 in ECC subjects, and 6.86 in S-ECC subjects. Statistical analysis showed that prevalence of ECC significantly increased with age (OR = 1.95; 95% CI = 1.3-2.91) and duration of breastfeeding (OR = 1.26; 95% CI = 1.01-1.57), whereas it was significantly lower in children of

			eligible children were invited to participate filling out a structured self-administered questionnaire, and after having returned the informed consent form an oral examination of the child was performed at school. The questionnaire included information on: socio-demographics about parents/guardians and child, pregnancy and newborn characteristics, oral hygiene habits of child, eating habits particularly on consumption of sweets, access to dental services, and infant feeding practices	more educated mothers (OR = 0.64; 95% CI = 0.42-0.96), and higher in those who had been visited by a dentist in the previous year (OR = 3.29; 95% CI = 1.72-6.33).
**A longitudinal study of dental caries risk among very young low SES children**	2009	Assess baseline risk factors for 18-month caries prevalence as part of a longitudinal study of high-risk children.	About 212 children, 6-24 months of age were recruited from a rural community in Iowa. Subjects were enrolled in the WIC program, which provides nutritional support for low-income families with children. Dental examinations using d1, d2-3 criteria were conducted at baseline and after 18 months. Caries prevalence was determined at the frank decay level (d2-3 or filled surfaces), as well as at the noncavitated level (d1), and combined (d1, d2-3 or f surfaces). Risk factor data were collected at baseline and after 9- and 18- months.	About 128 children (60%) remained in the study after 18 months. Among these children, prevalence of d1,d2-3/f level caries increased from 9% to 77%, while d2-3/f level caries increased from 2% to 20%. Logistic regression models for baseline predictors of d2-3f caries at the 18-month follow-up found the presence of MS in children (OR=4.4; 95% CI: 1.4, 13.9) and sugar-sweetened beverages (OR=3.0; 95% CI: 1.1, 8.6) to be the only significant risk factors.
**Longitudinal study of prolonged breast- or bottle-feeding on dental caries in Japanese children**	2006	Study the effects of prolonged breast- or bottle-feeding on dental caries in Japanese infants	his longitudinal study was conducted by means of a questionnaire and clinical examination of 592 children at 18	Results showed that breast-feeding at 18 months of age produced many significant differences to the control children, including a higher prevalence of caries and higher number of dft.

			months, 2 years and 3 years of age. The children were divided into three groups: 1) children still being breast-fed at 18 months of age (n=42); 2) children still being bottle-fed at 18 months of age (n=45); and 3) children weaned off of breast- or bottle-feeding and with no nonnutritive-sucking habits at 18 months of age (n=205).	However, no significant differences were observed between bottle-fed and control children. In conclusion, our results suggest that prolonged breast-feeding at an early age before primary dentition has fully erupted is a risk factor for dental caries.
**Association between mother-infant salivary contacts and caries resistance in children: a cohort study**	1994		There were selected 327 7-month-old infants and divided them into two groups based on the frequency of salivary close contacts between mother and infant. Five to seven years later, all first-born children (N = 55) whose dental development had been followed regularly, were examined for dental caries and prevalence of salivary mutans streptococci (MS) and lactobacilli.	The children with frequent maternal close contacts (F group, N = 21) had significantly less MS in saliva than the children with rare close contacts (R group, N = 34, P = 0.02). Only 19% of the children in F group compared with 56% in R group had experienced caries in their primary molars and/or canines (P < 0.01). A significantly greater proportion of the children in F group (57%) than in R group (27%, P < 0.05) had a high intake of sugar-containing foods and drinks in a 2-day dietary history. The F and R groups did not differ significantly with respect to other children's caries ris
**British Society of Paediatric Dentistry: a policy document on oral health care in preschool children**	2003	Prepare policy document	Policy documents produced by BSPD represent a majority view, based on a consideration of currently available evidence. They are produced to provide guidance, with the clear intention that the policy be regularly reviewed and updated to take account of changing views and developments.	
**Review of methods of identification of high caries risk groups and individuals. Fédération Dentaire Internationale Technical Report No. 31**	1988	Update the report on methods of caries prediction which resulted from the 1977 workshop, sponsored by the National Institutes of Health, Washington, DC, and also identify the methods most likely to provide effective prediction	The factors that need to be considered in assessing the value of a method of predicting caries risk are the correlation coefficient between the predictions and the final caries scores and in particular an assessment of the ability of the method to recognize subjects who will develop caries (sensitivity) and to	To date, a wide variety of factors have been considered in the search for an effective method of predicting caries risk, but only a few have had some success. Certain epidemiological methods have shown reasonable sensitivity but less specificity. Measures in this category include specific indicator surfaces and DMFT increment in the previous year. Among the more useful specific tests have been mutans streptococci and lactobacillus counts and measurement of saliva buffering capacity. Other methods that show

		of caries risk which should be given high priority in future research.	exclude those who will not (specificity). The predictive power of the method should also be known.	some promise include the physical measurement of incipient carious lesions of enamel.
**The dental health status of five-year-old children in north and west Belfast**	1997	Investigate the influence of social deprivation upon the diet, dental health behaviours and dental health status of five-year-old children in Northern Ireland	Dental health status measured by dmft index; parental assessments of the child's dental health behaviours: parental dental attendance patterns and attitudes towards dental health; parental assessments of the child's diet and snacking behaviours: parental attitudes towards snack foods; and demographic profile of child's family.	One hundred and sixty-three children were examined, a 68 per cent response. The majority of the families were either in low income employment or unemployed. Sixty-eight per cent of children had experience of dental caries. Dental decay was unrelated to parental employment status but more children from unemployed families attended when in pain. The diet of the children was related to both employment status and parental attitude and was reflected in their caries experience. Caries experience was dependent upon parental dental attendance, the consumption of carbonated drinks and sugar containing bedtime drinks.
**Risk factors of early childhood caries (ECC) among children in Beijing - a prospective cohort study**	2019	Explore the risk factors of ECC in Beijing, China.	Totle of 712 children (mean age: 46.37 ± 5.44 months) participated in this prospective cohort study. Questionnaires and the levels of mutans streptococci in dental plaques and non-stimulated saliva, assessed using Dentocult SM strips, were collected at baseline, respectively. Two calibrated examiners checked for dental caries according to the WHO 1997 criteria at baseline and 1-year follow-up. Negative binomial regression was used for multivariate analysis of factors related to caries development.	For caries-free children at baseline, only plaque mutans streptococci (PMS) levels were associated with caries development (odds ratio [OR] = 1.68, 95% confidence interval [CI]: 1.08–2.61, $P = 0.02$). Children with high PMS levels (scored 2 and 3) had higher caries incidence (46.2% vs. 33.8%) and more caries increment (1.87 ± 3.17 vs. 0.90 ± 1.89) than those with low levels (scored 0 and 1). Among all participants, PMS (OR = 1.69, 95% CI: 1.32–2.23, $P < 0.001$) and previous caries experience (OR = 2.80, 95% CI: 2.20–3.56, $P < 0.001$) were related to caries increment.
**Comparison of the salivary and dentinal microbiome of children with severe-early childhood caries to the salivary microbiome of caries-free children**	2019	Describe and compare the microbiota of 1) deep dentinal lesions of deciduous teeth of	Children with S-ECC and without S-ECC were recruited. The saliva of all children with and without S-ECC was sampled along with the	Using two beta diversity metrics (Bray Curtis dissimilarity and UniFrac distance), the caries microbiota was found to be distinct from that of either of the saliva groups (caries-free & caries-active) when bacterial abundance

		children affected with severe early childhood caries (S-ECC) and 2) the unstimulated saliva of these children and 3) the unstimulated saliva of caries-free children, and to compare microbiota compositional differences and diversity of taxa in these sampled sites.	deep dentinal microbiota from children affected by S-ECC. The salivary microbiota of children affected by S-ECC (n = 68) was compared to that of caries-free children (n = 70), by Illumina MiSeq sequencing of 16S rRNA amplicons. Finally, the caries microbiota of deep dentinal lesions of those children with S-ECC was investigated.	was taken into account. However, when the comparison was made by measuring only presence and absence of bacterial taxa, all three microbiota types separated. While the alpha diversity of the caries microbiota was lowest, the diversity difference between the caries samples and saliva samples was statistically significant ($p < 0.001$). The major phyla of the caries active dentinal microbiota were Firmicutes (median abundance value 33.5%) and Bacteroidetes (23.2%), with Neisseria (10.3%) being the most abundant genus, followed by Prevotella (10%). The caries-active salivary microbiota was dominated by Proteobacteria (median abundance value 38.2%) and Bacteroidetes (27.8%) with the most abundant genus being Neisseria (16.3%), followed by Porphyromonas (9.5%). Caries microbiota samples were characterized by high relative abundance of Streptococcus mutans, Prevotella spp., Bifidobacterium and Scardovia spp.
**Changes in dental caries 1953-2003**	2004	In the first half of the 20th century, indices and methods of conducting surveys of the level of dental diseases were developed. Modern epidemiological studies began in the fifties and many reliable studies have been conducted after 1960. In the following decades, a substantial decline of caries prevalence was documented in the majority of the highly industrialized countries, with reductions of lifetime caries experience exceeding 75%. The decline comes to an end when low or very low levels of prevalence are reached.		Children of low socioeconomic status and immigrants from outside Western Europe, however, generally have higher disease levels and may cause increases in caries prevalence.
**Social gradients and cumulative effects of income and education on dental health in the Fourth German Oral Health Study**	2010	Consider differential effects of income and education on oral health for each indicator separately and in combination. Finally the combined effects of the lowest income level and the lowest level of education were examined.	Data were drawn from the Fourth German Oral Health Study. They were collected using proportional random sampling in order to obtain information also for less densely populated regions. The subjects included in the study were between 35 and 44 years of age (n = 925). It included a clinical dental examination and a sociological survey.	Social gradients emerged for both indicators of social differentiation. The effects derived from single analyses were somewhat higher than those obtained by simultaneous estimations. The odds ratio of the lowest as compared with the highest income category was OR = 3.74 and OR = 2.34 in the analysis with both indicators. For education the respective effects were OR = 3.75 and OR = 2.95. The cumulative effect of the lowest income and the lowest educational level combined was OR = 6.06.

			Social differentiation was depicted by education and income (divided into categories), oral health was measured using the DMFT-index.	
**Caries in pre-school children in Camden 1993/94**	1996	Present survey were to determine the current prevalence of caries and of rampant caries and to relate prevalence to social factors, to infant feeding habits and to the use of sweetened comforters, and to determine the extent of dental care received.	Four hundred and six children were included in the current survey, which used the same sampling and methodology as previously.	Caries affected 11% of 2-year-old children and 29% of 3-year-olds, rampant caries affected 6% and 14% respectively. A higher likelihood of caries occurred in children of Asian origin and those given a sweetened comforter. For rampant caries, likelihood was greater amongst those of Asian origin and those given a sweet drink in a bottle for 2 years or longer.
**A prospective study on sucrose consumption, visible plaque and caries in children from 3 to 6 years of age**	2001	As data on the association of sugar consumption and dental caries in the industrialized countries give mixed results, we prospectively studied this association in 135 healthy Finnish children (71 boys, 64 girls).	The dental health and oral hygiene of the children was first examined at the mean age (+/-SD) of 37.4 (+/-2.1) months and again at 73.7 (+/-2.6) months. On both occasions the parents were interviewed about the child's sweet intake and toothbrushing habits, and sucrose consumption was analyzed using 4-day food diaries.	The proportion of children with caries experience, enamel and dentin lesions combined, increased from 16% to 40%. Daily sucrose intake of children who developed caries by 6 years of age, whether expressed as absolute (g) or as relative (E%) amounts, was already higher at 3 years of age than that of children who stayed caries-free ($P<0.05$ and $P<0.03$, respectively). Furthermore, children who used sweets more than once a week at 3 years of age, consumed more sucrose 3 years later ($P<0.01$) than those who used sweets once a week or less. The proportion of children with a combination of a sweet intake more than once a week and visible plaque, increased ($P<0.05$) during the follow-up. The risk ratio of children with the combined risk habit at 3 years of age to develop carious lesions by 6 years of age was 1.7 compared to the rest of the children (95% confidence interval 0.9-3.0).
**The fidelity of mutans streptococci transmission and caries status correlate with breast-feeding experience among Chinese families**	2000	Investigate those factors that could be correlated with MS transmission among Chinese	Forty-eight families with 2- to 3-year-old children were selected from two kindergartens of Beijing, China. A questionnaire	The data suggest that breast-feeding, especially prolonged breast-feeding, may correlate with the fidelity of transmission and that prolonged breast-

		children whose nurturing histories were known.	concerning the childhood nursing practice was obtained from the parents. Dental caries status was examined, and bacterial samples were collected for all participants twice at 6-month intervals. An average of six isolates of MS was picked at random from selective medium from each individual at each visit. Chromosomal DNA fingerprints were performed for all MS isolates to determine the genomic similarity within each family and among individuals.	feeding may contribute to a higher caries rate.
**The influence of enamel defects on the development of early childhood caries in a population with low socioeconomic status: a longitudinal study**	2006	Evaluate the influence of enamel defects in the development of dental caries and their association with feeding practices and oral health behaviors in a cohort study of low-socioeconomic children from birth to 36 months of age in northeastern Brazil.	Subjects were registered at birth and examined from 12 to 36 months of age. At baseline, 246 children were examined and at follow-up 228. The teeth were clinically examined and dried with gauze under natural light. Enamel defects were determined using the Developmental Defects of Enamel (DDE) index. Dental caries was determined using WHO criteria. Data were analyzed using descriptive and analytical techniques.	At 36 months 78.9% infants presented at least one tooth with enamel defects and 25% of the children had at least one decayed tooth. A total of 16.9% teeth with enamel defects had become decayed ($p = 0.0001$). Opacity with enamel hypoplasia was the defect most frequently associated with dental caries ($p = 0.001$). Only 0.9% of the teeth without enamel defects developed caries. Multivariate analyses revealed that enamel defects, night breast-feeding and poor oral hygiene habits were predictors of dental caries at 18 and 24 months ($p < 0.05$). Considering the risk factors evaluated at 30 months of age, the presence of enamel defects was the single predictor of caries development at 36 months ($p = 0.0001$)
**The contribution of life course determinants to early childhood caries: a 2-year cohort study**	2012	Investigate the risk factors for ECC in the life course of a cohort of 8-month-old children.	A total of 225 children aged 8 months were recruited from a rural community in the city of Guangzhou in southern China. Information was collected at baseline and once every 6 months. The questionnaire was designed to collect socioeconomic and behavioural information. Dental caries was	The incidence density among 8 to 14-, 14 to 20-, 20 to 26- and 26 to 32-month-old children was found to be 0, 0.02 ± 0.19, 0.05 ± 0.26 and 0.20 ± 0.59 (surfaces/100 surface-months), respectively. Multivariable analysis showed that mothers who had a low level of education, families with higher monthly income, enamel hypoplasia of the teeth, lower than average height, a high proportion of visible plaque, and the presence of S. mutans were risk predictors for ECC of the children.

			recorded by surface and oral hygiene was assessed using the visible plaque index. The presence of Streptococcus mutans (S. mutans) in dental plaque was determined using microbiological tests. The incidence density of ECC was calculated and generalised estimating equation analysis was performed to identify the risk factors.	
Association between Enamel Hypoplasia and Dental Caries in Primary Second Molars: A Cohort Study	2009	Assess the longitudinal relationships between enamel hypoplasia and caries experience of primary second molars	The study sample was 491 subjects who received dental examinations at both age 5 and 9 by the calibrated examiners. Four primary second molars (n = 1,892) were scored for the presence of enamel hypoplasia for each participant. Caries presence and number of decayed and filled surfaces (dfs) were determined at age 5 and 9.	The relationships between enamel hypoplasia and caries experience were assessed. Among primary second molars, 3.9% of children and 1.7% of primary second molars had enamel hypoplasia. At age 5, 36.8% of children with hypoplasia had caries, while 16.9% of children without enamel hypoplasia had caries. At age 9, the corresponding numbers were 52.6% for children with hypoplasia and 34.5% for children without hypoplasia, respectively. At the tooth level, for age 5, 28.1% of teeth with hypoplasia had caries (mean dfs = 0.40), and 7.6% of teeth without hypoplasia had caries (mean dfs = 0.11). At age 9, the corresponding numbers were 41.9% (mean dfs = 0.76) for teeth with hypoplasia and 18.3% (mean dfs = 0.34) for teeth without hypoplasia. In multivariable logistic regression analyses, teeth of subjects with enamel hypoplasia had a significantly higher risk for caries at age 5 and 9 after controlling for other risk factors.
**Case-control study of early childhood caries in Australia**	2009	Investigate early childhood caries (ECC) risk indicators in a non-fluoridated region in Australia.	ECC cases were recruited from childcare facilities, public hospitals and private specialist clinics to source children from different socioeconomic backgrounds. Non-ECC controls were recruited from the same childcare facilities. A multinomial logistic modelling	The results showed that a large percentage of children tested positive for Streptococcus mutans if their mothers also tested positive. A common risk indicator found in ECC children from childcare facilities and public hospitals was visible plaque (OR 4.1, 95% CI 1.0-15.9, and OR 8.7, 95% CI 2.3-32.9, respectively). Compared to ECC-free controls, the risk indicators specific to childcare cases were enamel hypoplasia (OR 4.2, 95% CI 1.0-18.3),

			approach was used for statistical analysis.	difficulty in cleaning child's teeth (OR 6.6, 95% CI 2.2-19.8), presence of S. mutans (OR 4.8, 95% CI 0.7-32.6), sweetened drinks (OR 4.0, 95% CI 1.2-13.6) and maternal anxiety (OR 5.1, 95% CI 1.1-25.0). Risk indicators specific to public hospital cases were S. mutans presence in child (OR 7.7, 95% CI 1.3-44.6) or mother (OR 8.1, 95% CI 0.9-72.4), ethnicity (OR 5.6, 95% CI 1.4-22.1), and access of mother to pension or health care card (OR 20.5, 95% CI 3.5-119.9). By contrast, a history of chronic ear infections was found to be protective for ECC in childcare children (OR 0.28, 95% CI 0.09-0.82).
**The relationship of enamel defects and caries: a cohort study**	2011	Assess relationship between enamel defects and early childhood caries	A total of 275 children participated in a cohort study from birth to 54 months of age. Enamel defects were determined by the development defects enamel index and dental caries was registered according to the WHO criteria. Data were analyzed using descriptive, analytical techniques, multivariate analysis, and evidence-based tools as number needed to harm (NNH).	In the follow up, 224 children were still in the study, 81.3% presented at least one tooth with enamel defect and 44.2% had dental caries. An association was found between enamel defects and dental caries ($P = 0.0091$). Multivariate analysis showed that night bottle-feeding, absence of fluoride and enamel defects were predictors of dental caries at 18 months ($P < 0.05$). Enamel defect was the only statistically significant variable to influence the development of caries at 24, 30, 36, and 42 months. At 48 months, the use of fluoride toothpaste had effect on the decrease of caries ($P < 0.05$). The NNH for enamel defects in relation to dental caries was 3.0, at 24 months and 5.0 at 54 months.
**Enamel hypoplasia and dental caries in Australian aboriginal children: prevalence and correlation between the two diseases**	1994	Investigate the prevalence of enamel hypoplasia and dental caries and the relationship between the two diseases in all 4- to 6-year-old Australian Aboriginal children of the Tiwi tribe on Bathurst Island	Seventy-nine of 80 children (99%) had enamel hypoplasia, with a mean of 12.0 +/- 4.1 hypoplastic teeth per child. Dental caries was noted in 66 (83%) of children and the mean number of decayed teeth per child was 3.9 +/- 3.3.	A strong association between enamel hypoplasia and dental caries ($P < 0.01$) suggests that enamel hypoplasia may be a significant caries risk factor in this group. Furthermore, while high levels of medical morbidity were found, the relative importance of each medical condition in the pathogenesis of enamel hypoplasia could not be determined because nearly all patients with enamel hypoplasia had the full range of medical problems
**Feeding practices and dental caries in an urban Canadian population of Vietnamese preschool children**	1997	Determine the severity of nursing caries, and to examine	The data collected became the basis for a community-based oral health promotion	Sixty-five percent of all children had a naptime bottle, and 85 percent > or = 18 mos had a "comfort" bottle that was carried around, and drunk from during

		contributing behavioral factors, in a group of Vietnamese families in British Columbia, Canada	program. Information on feeding, dental health practices, and dental caries were collected for 60 mother/child pairs.	the day. Milk was the most common beverage. A "comfort" bottle was significantly related to the presence of nursing caries, P = 0.02; a naptime bottle had a less significant association, P = 0.07. Dental knowledge questions revealed that all mothers knew that a child who had a "comfort" bottle could get tooth decay, but 63 percent thought that cavities were not a problem in baby teeth.
**Occurrence of dental decay in children after maternal consumption of xylitol chewing gum, a follow-up from 0 to 5 years of age**	2000	Show that prevention of mutans streptococci (MS) colonization in early childhood can lead to prevention of dental decay. In the microbiological part	The children were examined annually for caries occurrence by experienced clinicians who did not know whether the children were colonized with MS.	In children at the age of 5 years, the dentinal caries (dmf) in the xylitol group was reduced by about 70% as compared with that in the fluoride or chlorhexidine group. We conclude that maternal use of xylitol chewing gum can prevent dental caries in their children by prohibiting the transmission of MS from mother to child.
**Socioeconomic status and risk of dental caries in Japanese preschool children: the Osaka Maternal and child health study**	2013	Examine the impact of socioeconomic factors, such as maternal occupation, household income, and parental educational levels, on the risk of dental caries in young Japanese children.	A cohort of 315 preschool children was used. Information pertaining to exposure and potentially confounding factors was obtained by means of questionnaires administered to expectant mothers prior to delivery and subsequently when their children were in the ranges of 2-9, 16-24, 29-39, and 41-49 months of age. Outcome data were collected when the children were between 41 and 50 months old. Children were classified as having dental caries if one or more primary teeth had decayed or had been filled.	Compared with maternal or paternal education of less than 13 years, maternal or paternal education of 15 years or longer was significantly associated with a decreased risk of dental caries in children: the adjusted odds ratios for maternal and paternal education levels ≥ 15 years compared with <13 years of maternal and paternal education were 0.32 (95 percent confidence interval: 0.14-0.71) and 0.45 (95 percent confidence interval: 0.23-0.88), respectively. No relationship between maternal occupation or household income and the risk of children's dental caries was found.
**Prevalence of dental caries and associated social risk factors among preschool children in Riyadh, Saudi Arabia**	2016	Determine the prevalence of dental caries, and associated social risk factors among preschool children in Riyadh, Saudi Arabia.	The study consisted of a random sample of 3 to 5 years- old preschool children who were examined in Riyadh, Saudi Arabia; 388 children (184 boys and 204 girls) were examined	About 69% of children had dental caries with dmft score of 3.4 (± 3.6) and dmfs of 6.9 (± 9.9). There was no statistically significant difference between boys and girls. Less caries was observed among children whose parents worked and it was statistically significant as well as whose mothers had high or low

			from 10 different preschools. Each surface of their teeth was examined for dental caries utilizing modified WHO criteria (WHO, 1997). Data information about age, gender and social factors status were obtained by questionnaires that had been answered by parents.	educational level. Increased number of family members appeared to have a high incidence of dental caries which was also statistically significant. There was no significant difference in dental caries prevalence with birth order.
**Parental occupations, educational levels, and income and prevalence of dental caries in 3-year-old Japanese children**	2017	Examine the associations between parental occupations and levels of education and household income and the prevalence of dental caries in Japanese children aged 3 years.	Study subjects were 6315 children. Oral examination results were obtained from the parents or guardians, who transcribed the information recorded by medical staff at a public health center from their maternal and child health handbooks to our self-administered questionnaire. Children were classified as having dental caries if one or more primary teeth had decayed or had been filled. Adjustment was made for sex, age, region of residence, breastfeeding duration, between-meal snack frequency, toothbrushing frequency, use of fluoride, regular dental check-ups, maternal smoking during pregnancy, and living with at least one household smoker.	The prevalence of dental caries was 14.7%. Compared with having an unemployed father, having a father employed in professional and engineering, clerical, sales, security, or manufacturing process was significantly associated with a lower prevalence of dental caries. Compared with having an unemployed mother, having a mother employed in professional and engineering or service was significantly inversely associated with the prevalence of dental caries. Significant inverse associations were observed between parental levels of education and household income and the prevalence of dental caries.
